# Advanced care planning during the COVID-19 pandemic: ceiling of care decisions and their implications for observational data

**DOI:** 10.1186/s12904-021-00711-8

**Published:** 2021-01-11

**Authors:** Sam Straw, Melanie McGinlay, Michael Drozd, Thomas A. Slater, Alice Cowley, Stephe Kamalathasan, Nicholas Maxwell, Rory A. Bird, Aaron O. Koshy, Milos Prica, Peysh A. Patel, Samuel D. Relton, John Gierula, Richard M. Cubbon, Mark T. Kearney, Klaus K. Witte

**Affiliations:** 1 Leeds Institute of Cardiovascular and Metabolic Medicine, University of Leeds, Leeds, UK; 2 Leeds Teaching Hospitals NHS Trust, Leeds, UK; 3 School of Medicine, University of Leeds, Leeds, UK; 4 Leeds Institute of Health Sciences, University of Leeds, Leeds, UK

**Keywords:** COVID-19, Resuscitation, Advanced care planning, Comorbidity, Elderly, Geriatrics

## Abstract

**Background:**

Observational studies investigating risk factors in coronavirus disease 2019 (COVID-19) have not considered the confounding effects of advanced care planning, such that a valid picture of risk for elderly, frail and multi-morbid patients is unknown. We aimed to report ceiling of care and cardiopulmonary resuscitation (CPR) decisions and their association with demographic and clinical characteristics as well as outcomes during the COVID-19 pandemic.

**Methods:**

Retrospective, observational study conducted between 5th March and 7th May 2020 of all hospitalised patients with COVID-19. Ceiling of care and CPR decisions were documented using the Recommended Summary Plan for Emergency Care and Treatment (ReSPECT) process. Unadjusted and multivariable regression analyses were used to determine factors associated with ceiling of care decisions and death during hospitalisation.

**Results:**

A total of 485 patients were included, of whom 409 (84·3%) had a documented ceiling of care; level one for 208 (50·9%), level two for 75 (18·3%) and level three for 126 (30·8%). CPR decisions were documented for 451 (93·0%) of whom 336 (74·5%) were ‘not for resuscitation’. Advanced age, frailty, White-European ethnicity, a diagnosis of any co-morbidity and receipt of cardiovascular medications were associated with ceiling of care decisions. In a multivariable model only advanced age (odds 0·89, 0·86–0·93 *p* < 0·001), frailty (odds 0·48, 0·38–0·60, *p* < 0·001) and the cumulative number of co-morbidities (odds 0·72, 0·52–1·0, *p* = 0·048) were independently associated. Death during hospitalisation was independently associated with age, frailty and requirement for level two or three care.

**Conclusion:**

Ceiling of care decisions were made for the majority of patients during the COVID-19 pandemic, broadly in line with known predictors of poor outcomes in COVID-19, but with a focus on co-morbidities suggesting ICU admission might not be a reliable end-point for observational studies where advanced care planning is routine.

## Background

In December 2019, severe acute respiratory syndrome coronavirus 2 (SARS-CoV-2) was first identified as the causative agent for a cluster of pneumonia cases in Wuhan City, China [1]. A global pandemic was declared by the World Health Organisation (WHO) in March 2020, which continues to challenge healthcare systems globally. SARS-CoV-2 can result in a spectrum of illness, from asymptomatic infection to coronavirus disease 2019 (COVID-19), a viral pneumonia resulting in high rates of hospitalisation, intensive care unit (ICU) admission and requirement for mechanical ventilation [2, 3]. There has been considerable interest in exploring risk factors for susceptibility to infection and severe disease in COVID-19, with proposed risk factors including age [4], cardiovascular co-morbidities (including diabetes mellitus) [5], medications inhibiting the renin-angiotensin-aldosterone system (RAAS) [6], obesity [7] and ethnicity [8].

Observational data have resulted in contradictory findings, possibly owing to heterogeneous definitions of what constitutes severe disease, with hospitalisation, ICU admission [3, 9, 10], mechanical ventilation [11] or death [12, 13] explored as primary end-points. Despite this, little is known about how decisions to limit care below full intensive care-based treatments were made during the COVID-19 pandemic.

The aims of this analysis were therefore firstly, to report pre-emptive ceiling of care and cardiopulmonary resuscitation (CPR) decisions in patients admitted with SARS-CoV-2 infection in a large university teaching hospital trust in the UK during the COVID-19 pandemic. Secondly, we aimed to describe the demographic and clinical characteristics associated with ceiling of care decisions, along with reporting their clinical outcomes.

## Methods

### Patients

We performed a retrospective, observational study to explore factors associated with outcomes in COVID-19 at the Leeds Teaching Hospitals NHS Trust (LTHT), one of the largest university teaching hospitals in Europe, comprising of two large and four smaller facilities providing over 1800 inpatient beds, serving a secondary care population of more than 750,000 people. All patients aged ≥18 years with laboratory confirmed SARS-CoV-2 infection hospitalised at LTHT between 5th March and 7th May 2020 were included. Approval was given following institutional governance review and, in view of the retrospective nature, individual patient consent was waived as appropriate data protection safeguards were in place. In keeping with WHO guidance, laboratory confirmation for SARS-CoV-2 was defined as a positive result of real-time reverse transcriptome-polymerase chain reaction assay of nasal or pharyngeal swabs, or lower respiratory tract aspirates [14]. Patients who tested positive, but admitted for other reasons or were infected with SARS-CoV-2 during hospitalisation were excluded, as were those who were assessed in the Emergency Department but not hospitalised.

### Data sources and definitions

Clinical data and outcomes were obtained from the Leeds Patient Pathway Manager Plus electronic care record, which updates mortality events daily directly from the UK Office of National Statistics database. Ceiling of care and CPR decisions were standardised and documented electronically using the Recommended Summary Plan for Emergency Care and Treatment (ReSPECT) process (Resuscitation Council UK) [15]. Demographic data include age, sex and ethnicity. Ethnicity was self-reported and classified according to the 2011 Census for England, Northern Ireland and Wales as White-European, South-Asian, East-Asian, Black-African, mixed race and other ethnicities, and, for the purpose of analysis, was dichotomised as White-European or Black, Asian and minority ethnic (BAME). Clinical data include major co-morbidities, frailty and the prescription of medical therapy. Major co-morbidities were any of: hypertension, diabetes mellitus, chronic kidney disease (CKD) stage III-V, atrial fibrillation, chronic obstructive pulmonary disease (COPD), ischaemic heart disease (IHD), heart failure with reduced ejection fraction (HFrEF), history of stroke or transient ischaemic attack (TIA) and active malignancy. Frailty was classified by the Canadian Study of Health and Aging Clinical Frailty Scale (CFS) [16] according to national recommendations [17], which, during the COVID-19 pandemic, was mandatory for all patients assessed in the Emergency Department at LTHT. Pre-admission medical therapy was obtained by regular prescription information from electronic primary care records. We recorded the prescription of RAAS inhibitors, beta-blockers, calcium channel blockers, diuretics, statins, antiplatelet and anticoagulants, medications for diabetes mellitus and immunosuppression. We recorded clinical markers of disease severity at the time of hospitalisation, including laboratory investigations, chest radiography and clinical observations. Laboratory investigations included full blood count, renal function and blood tests to stratify disease severity, which were C-reactive protein, ferritin, D-dimer and procalcitonin. All chest radiographs were interpreted by a radiologist and graded as either being consistent with, indeterminant for, or inconsistent with COVID-19 pneumonia. Clinical observations included heart rate, blood pressure, tympanic temperature, peripheral oxygen saturations and respiratory rate, and were obtained from the earliest assessment of physiology, usually recorded by paramedic crew or on arrival in the Emergency Department.

### Assessment of outcomes

Patients were followed-up until discharge from hospital or death. Outcomes data include treatments administered during hospitalisation, maximum level of care received, and death prior to discharge. Administered treatments were oxygen therapy, continuous positive pressure ventilation (CPAP), mechanical ventilation, circulatory support (vasopressors or inotropes) or new requirement for renal replacement therapy. Level one care was hospitalisation without need for organ support (but including oxygen therapy) and delivered in a ward setting; level two care was single organ support (usually CPAP), but excluding mechanical ventilation, and delivered in either in a ward, high-dependency unit or ICU setting; level three care was multi-organ support or mechanical ventilation and delivered on the ICU.

### Statistical analysis

All statistical analyses were performed using IBM SPSS Statistics version 26 (IBM Corporation, Armonk, NY). After testing for normality of distribution, continuous variables are expressed as mean ± standard deviation or median (interquartile range), as appropriate. Discrete variables are presented as number (percentage), and ordinal data as median (interquartile range). Groups were compared using Student’s t-test or one-way analysis of variance for normally distributed continuous data, by Mann-Whitney U test or Kruskal-Wallis H-test for non-normally distributed continuous data and by Pearson χ^2^ tests for categorical data. Age-sex adjusted and multivariable analyses were performed using binary logistic regression analysis. All tests were two-sided and statistical significance was defined as *p* < 0·05.

## Results

### Patients

Between 5th March and 7th May 2020, a total of 599 patients tested positive for SARS-CoV-2 in LTHT and of these, 65 were admitted for reasons other than COVID-19, 38 were not hospitalised, five were aged < 18 years and six tests were subsequently amended as negative following quality control. The final dataset therefore consisted of 485 patients, with a mean age of 71.2 ± 16.9 years of whom 259 (53·4%) were male. A total of 109 (22·5%), 130 (26·8%), 105 (21·6%) and 141 (29·1%) had zero, one, two and three or more major co-morbidities, respectively. The most common co-morbidity was hypertension, which was present in 222 (45·8%) patients, whilst 147 (30·3%) had diabetes mellitus (Table 1). Self-reported ethnicity was available for 475 patients (97·9%), of whom 402 (84·6%) classified themselves as White-European, 31 (6·5) as South-Asian, 19 (4·0%) as Black-African, two (0·4%) as East-Asian and 21 (4·4%) as either mixed or other ethnicities.

**Table 1:** Baseline clinical characteristics of patients divided by ceiling of care decisions

	All patients (*n* = 409)	Level 1 (*n* = 208)	Level 2 (*n* = 75)	Level 3(*n* = 126)	*p-*value
Demographics					
Age (years)	73·1 ± 15·3	81·9 ± 9·4	75·4 ± 9·9	57·6 ± 12·8	**< 0·001**
Male sex [n(%)]	211 (54)	103 (49·5)	43 (57·3)	75 (59·5)	0·17
BMI (kg/m^2^)	26·3 (22·1–30·8)	23·2 (20·5–27·1)	27·6 (22·0–31·8)	29·4 (25·8–34·0)	**< 0·001**
BAME [n(%)]	56 (13·7)	12 (5·8)	8 (10·7)	36 (28·6)	**< 0·001**
Clinical Frailty Scale	5 (3–6)	6 (5–7)	4 (3–5)	2 (2–3)	**< 0·001**
Co-morbidities					
HFrEF [n(%)]	51 (12·5)	34 (16·3)	13 (17·3)	4 (3·2)	**0·001**
IHD [n(%)]	62 (15·2)	39 (18·8)	15 (20·0)	8 (6·3)	**0·004**
Hypertension [n(%)]	191 (46·7)	99 (47·6)	44 (58·7)	48 (38·1)	**0·017**
AF [n(%)]	87 (21·3)	60 (28·8)	17 (22·7)	10 (7·9)	**< 0·001**
Diabetes mellitus [n(%)]	125 (30·6)	67 (32·2)	32 (42·7)	26 (20·6)	**0·004**
Stroke/TIA [n(%)]	48 (11·7)	34 (16·3)	10 (13·3)	4 (3·2)	**0·001**
CKD [n(%)]	103 (25·2)	69 (33·2)	29 (38·7)	5 (4·0)	**< 0·001**
COPD [n(%)]	64 (15·6)	41 (19·7)	16 (21·3)	7 (5·6)	**0·001**
Malignancy [n(%)]	33 (8·1)	22 (10·6)	7 (9·3)	4 (3·2)	**0·050**
Medications					
ACEi [n(%)]	74 (18·1)	30 (14·4)	23 (30·7)	21 (16·7)	**0·007**
ARB [n(%)]	32 (7·8)	10 (4·8)	8 (10·7)	14 (11·1)	0·069
BB [n(%)]	99 (24·2)	61 (29·3)	22 (29·3)	16 (12·7)	**0·001**
CCB [n(%)]	68 (16·6)	27 (13·0)	20 (26·7)	21 (16·7)	**0·024**
Loop diuretic [n(%)]	64 (15·6)	50 (24·0)	12 (16·0)	2 (1·6)	**< 0·001**
MRA [n(%)]	16 (3·9)	11 (5·3)	3 (4·0)	2 (1·6)	0·24
Statin [n(%)]	171 (41·8)	85 (40·4)	49 (65·3)	38 (30·2)	**< 0·001**
Antiplatelet [n(%)]	105 (25·7)	57 (27·4)	23 (30·7)	25 (19·8)	0·17
Anticoagulant [n(%)]	57 (13·9)	42 (20·2)	11 (14·7)	4 (3·2)	**< 0·001**
Metformin [n(%)]	50 (12·2)	23 (11·1)	14 (18·7)	13 (10·3)	0·17
Sulphonylurea [n(%)]	20 (4·9)	5 (2·4)	8 (10·7)	7 (5·6)	**0·016**
Corticosteroid [n(%)]	21 (5·1)	12 (5·8)	3 (4·0)	6 (4·8)	0·82
Immunosuppression [n(%)]	21 (5·1)	9 (4·3)	5 (6·7)	7 (5·6)	0·71

*BMI* body mass index, *BAME* Black Asian and minority ethnic, *BMI* body mass index, *HFrEF* heart failure with reduced ejection fraction, *IHD* ischaemic heart disease, *AF* atrial fibrillation, *CKD* chronic kidney disease, *COPD* chronic obstructive pulmonary disease, *ACEi* angiotensin converting enzyme inhibitor, *ARB* angiotensin II receptor blocker, *BB* beta-adrenoceptor antagonist, *CCB* calcium channel blocker, *MRA* mineralocorticoid receptor antagonist

### Ceiling of care and cardiopulmonary resuscitation decisions

**Fig. 1:**
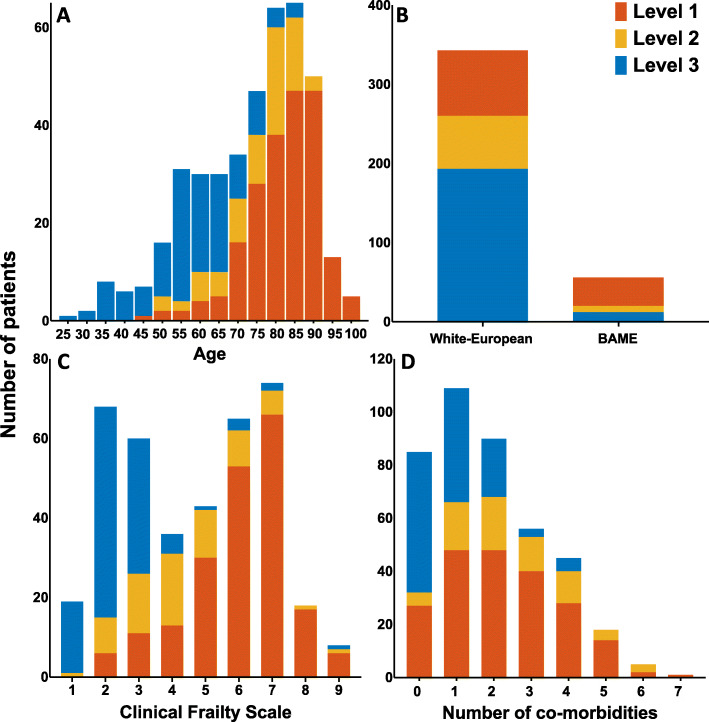
Bar charts showing **a** age, **b** ethnicity, **c** Clinical Frailty Scale and **d** co-morbidities in patients deemed appropriate for level one, two or three care. Patients in the present study were often elderly, frail and were multi-morbid

Bar charts showing ceiling of care decisions divided by patient demographics are displayed in Fig. 1. Following consultation with patients, their next-of-kin and surrogate decision makers, pre-emptive ceiling of care decisions were documented for 409 (84·3%) patients hospitalised with SARS-CoV-2 infection. Of patients in whom these decisions were made, 208 (50·9%), 75 (18·3%) and 126 (30·8%) patients were deemed suitable for a maximum of level one, two or three care, respectively. CPR decisions were made for 451 (93·0%) patients, of whom 336 (74·5%) were deemed not for CPR in event of cardiac arrest, with CPR deemed appropriate in 115 (25·5%).

### Demographics and clinical characteristics

**Fig. 2:**
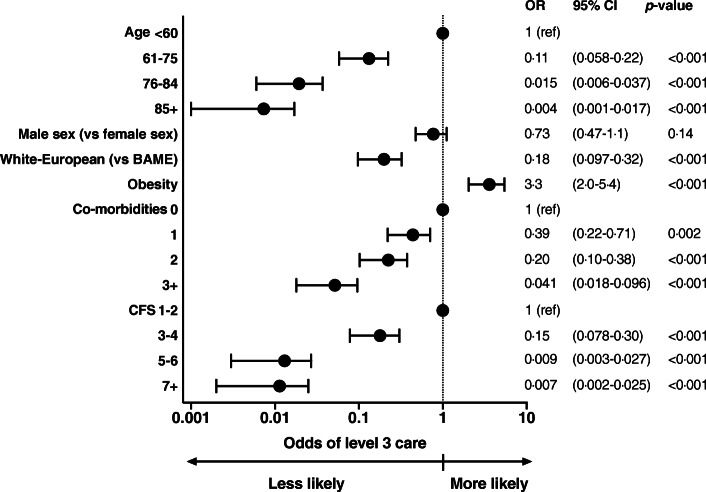
Forrest plot showing unadjusted odds ratios of appropriateness of level three care associated with demographic and clinical variables. There were associations between treatment escalation decisions and age, frailty and burden of co-morbidities

Patients considered suitable for escalation of treatment were younger, less frail and had fewer major co-morbidities. In unadjusted analysis, age was strongly associated with treatment escalation decisions, most evident in patients over 85 years of age (odds ratio (OR) 0·004, 95% confidence interval (CI) 0·001–0.017, *p* < 0·001). Other variables associated with ceiling of care decisions were higher CFS, lower BMI, a diagnosis of any major co-morbidity, the prescription of cardiovascular medications and White-European ethnicity (Fig. 2).

Compared to White-European patients, BAME patients were on average younger (58·0 ± 15·4 vs 73·7 ± 16.0 years, *p* < 0·001), had fewer major co-morbidities (1 (0,2) vs 2 (1, 3), *p* = 0·037) and were less frail (CFS 2 (2, 4) vs 5 (3, 7), *p* < 0·001). When adjusted for age and sex, ethnicity was not associated with ceiling of care decisions, nor were there associations between ceiling of care decisions and lower BMI or the prescription of most cardiovascular medications. Associations between a ceiling of care decision of less than level three and frailty, a diagnosis of diabetes mellitus, COPD, CKD, history of stroke or TIA and prescription of loop diuretic or statin remained when adjusted for age and sex. In multivariable regression analysis, predictors of ceiling of care decisions were advanced age (OR 1·1 per year, 95% CI 1·1–1·2, *p* < 0·001) and higher CFS (OR 2·1, 95% CI 1·7–2·7, *p* < 0·001) (Table 2). No other clinical or demographic variables were independently associated with the decision to limit the maximal care level provided. No individual co-morbidities featured as part of a multivariable analysis although there was a significant association between the cumulative number of major co-morbidities and ceiling of care decisions (OR 1·4 per co-morbidity, 95% CI 1·0–1·9, *p* = 0·048) (Table 3).

**Table 2:** Multivariable binary regression analysis of clinical characteristics and ceiling of care decisions (with individual co-morbidities)

	Odds ratio	95% CI	*p-*value
Age (per year)	0·89	0·86–0·92	**< 0·001**
Male sex	1·8	0·81–4·0	0·15
CFS (per nodal point)	0·47	0·37–0·60	**< 0·001**
Diabetes	0·49	0·20–1·2	0·11
COPD	0·60	0·19–1·9	0·38
CKD	0·31	0·093–1·1	0·062
Stroke/TIA	1·1	0·25–5·1	0·88
Loop diuretic	0·27	0·031–2·3	0·23
Statin	0·68	0·30–1·5	0·35

*CI* confidence interval, *CFS* Clinical Frailty Scale, *COPD* chronic obstructive pulmonary disease, *CKD* chronic kidney disease, *TIA* transient ischaemic attack

**Table 3:** Multivariable binary regression analysis of clinical characteristics and ceiling of care decisions (with cumulative number of co-morbidities)

	Odds ratio	95% CI	*p-*value
Age (per year)	0·89	0·86–0·93	**< 0·001**
Male sex	1·75	0·79–3·8	0·17
CFS (per nodal point)	0·48	0·38–0·60	**< 0·001**
Co-morbidities (per co-morbidity)	0·72	0·52–1·0	**0·048**
Loop diuretic	0·23	0·026–2·1	0·19
Statin	0·65	0·30–1·4	0·28

*CI* confidence interval, *CFS* Clinical Frailty Scale

### Clinical markers of disease severity

Patients deemed inappropriate for level three care had on average fewer markers of severe SARS-CoV-2 infection at the time of presentation compared to those who were (Table 4). Laboratory markers of systemic inflammation such as C-reactive protein and serum ferritin were more often abnormal in patients deemed eligible for escalation to level three care, as were assessments of physiology such as respiratory rate, heart rate and tympanic temperature. Chest radiography data were available for 471 (97·1%) patients at the time of hospitalisation of which 217 (44·7%) were reported as consistent with, 151 (31·1%) were indeterminate for and 103 (21·2%) inconsistent with COVID-19. Patients who were considered appropriate for level three care were more likely to have chest radiography consistent with COVID-19 compared to those who were not (*p* < 0·001), suggesting a higher severity of disease in these patients.

**Table 4:** Markers of severity of disease divided by ceiling of care decisions

	All patients (*n* = 409)	Level 1 (*n* = 208)	Level 2 (*n* = 75)	Level 3 (*n* = 126)	*p-*value
Laboratory findings					
Hb	129·2 ± 20·7	126·6 ± 21·8	125·0 ± 21·6	135·9 ± 16·5	**< 0·001**
WCC	7·0 (5·4–9·4)	7·2 (5·4–10·0)	6·5 (5·0–9·1)	7·1 (5·6–9·5)	0·23
ANC	5·4 (4·0–8·0)	5·7 (4·0–8·9)	5·1 (3·9–7·8)	5·6 (4·2–8·3)	0·38
Lymphocyte count	0·8 (0·5–1·1)	0·7 (0·5–1·1)	0·7 (0·5–1·0)	0·8 (0·6–1·1)	0·12
Na^2+^	138 (136–141)	140 (136–145)	137 (135·8–140)	137 (135–139)	**< 0·001**
K^+^	4·0 (3·7–4·4)	4·0 (3·7–4·5)	4·2 (3·7–4·5)	3·9 (3·7–4·2)	**0·034**
Creatinine	81 (63–117)	95 (68–143)	86·5 (65·8–129·5)	74 (60·3–89·8)	**< 0·001**
CRP	90 (45–169)	77·5 (34·8–159·8)	106 (72·3–187·3)	110 (68–192)	**0·001**
Ddimer	467 (258·5–1035·3)	496 (266–1978)	671 (387–1042)	373 (223–924)	0·068
hsTNI	20·5 (8·1–60)	40·0 (17·2–94·3)	25·5 (9·9–70·5)	8·5 (4·6–22·2)	**< 0·001**
Ferritin	460 (220–982)	365 (143·8–671·5)	510 (261·3–946)	674 (338·5–1458)	**< 0·001**
Procalcitonin	0·15 (0·08–0·38)	0·17 (0·08–0·52)	0·14 (0·08–0·32)	0·15 (0·09–0·40)	0·60
Clinical observations					
RR (min^−1^)	22 (18–28)	20 (18–28)	22·5 (20–28)	24 (20–29)	**0·026**
O_2_ saturations (%)	94·5 (89–96)	95 (90–97)	94 (88–96)	94 (89–96)	0·065
Heart rate (min^−1^)	90 (76–103)	89 (73–102·8)	89 (79–101·3)	96 (86–108·5)	**0·001**
SBP (mmHg)	129·2 ± 23·5	130·4 ± 25·5	131·2 ± 24·4	126·2 ± 18·8	0·21
Temperature (°C)	37·7 ± 1·1	37·4 ± 1·1	37·7 ± 1·1	38·0 ± 1·0	**< 0·001**
Chest radiography					
COVID-19 [n(%)]	190 (47·4)	61 (29·6)	40 (55·6)	89 (72·4)	**< 0·001**
Indeterminate [n(%)]	125 (31·2)	79 (38·2)	22 (30·6)	24 (19·5)	
Non-COVID-19 [n(%)]	86 (21·4)	66 (32·0)	10 (13·9)	10 (8·1)	

*Hb* haemoglobin, *WCC* white cell count, *ANC* absolute neutrophil count, *Na*^*2+*^ sodium, *K*^*+*^ potassium, *CRP* C-reactive protein, *hsTNI* high-sensitivity troponin-I, *RR* respiratory rate, *O*_*2*_ oxygen, *SBP* systolic blood pressure

### Treatments administered during hospitalisation

During hospitalisation, a total of 383 (79·0%) patients required oxygen therapy, 88 (18·1%) received CPAP, 38 (7·8%) received mechanical ventilation, renal replacement therapy was used in 11 (2·3%) and 28 (5·8%) patients required inotropes or vasopressors. CPAP was delivered in a ward setting for 6 (6.8%), on the high-dependency unit for 13 (14·8%) and on ICU for 69 (78·4%). All patients who required mechanical ventilation were cared for in an ICU setting. Overall, 92 (19·0%) of patients were admitted to ICU, 61 (81·3%) of whom were deemed suitable for level three care, whilst 14 (18·7%) were suitable for, and received, level two care.

### Clinical outcomes

**Fig. 3:**
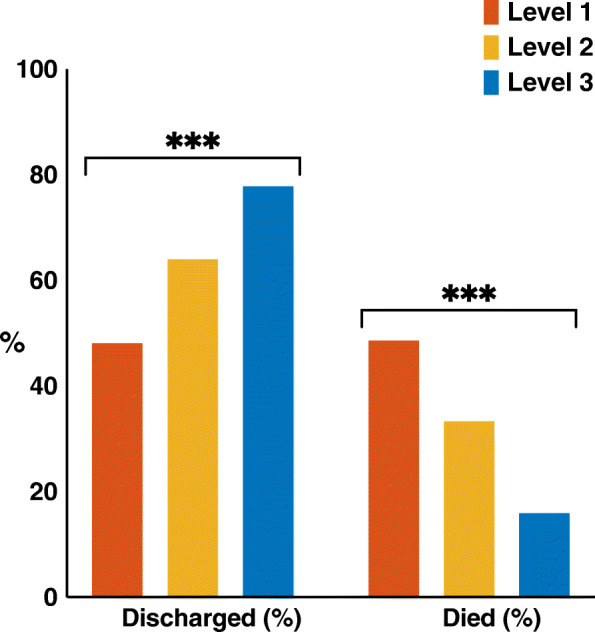
Bar charts showing outcomes of patients appropriate for level one, two or three care. Patients appropriate for level three care were the most likely to be discharged and least likely to have died during admission. *p*-value < 0·05*, < 0·01**, < 0·001***

At the time of censorship a total of 307 (63·3%) patients had been discharged from hospital following a mean hospital stay of 12·7 ± 10·5 days. Overall 159 (32·8%) patients died prior to discharge with a mean follow-up of 12·6 ± 11·2 days after admission, whilst 19 (3·9%) remained in hospital. Despite on average having more markers of disease severity, patients deemed to be suitable for level three care were more likely to be discharged and less likely to have died during hospitalisation (*p* < 0·001) (Fig. 3). Of the 20 (16%) patients eligible for level three care who died during the study period, all were admitted to ICU and received mechanical ventilation prior to death. Overall, including patients in whom ceiling of care decisions were not documented, 38 (7·8%) received mechanical ventilation during the study period and of these seven (18·4%) had been discharged, nine (23·7%) remained in hospital and 22 (57·9%) had died.

Death during admission was associated with advanced age, White-European ethnicity, higher CFS, a diagnosis of HFrEF, atrial fibrillation, CKD or COPD and the prescription of anticoagulant (Table 5). When adjusted for age and sex, associations between death during admission and higher CFS remained (OR 1·2, 95% CI 1·1–1·4, *p* = 0·001), but not the associations with White-European ethnicity, any major co-morbidity or medication. In a multivariable model including age, CFS and maximum level of care receiving during admission, receipt of level two (OR 2·6, 95% CI 1·1–11·6, *p* = 0·033) or level three care (OR 8·1, 95% CI 3·7–17·8, *p* < 0·001) were associated with an increased risk of death during hospitalisation.

**Table 5:** Baseline clinical characteristics of patients divided by those who were alive or had died during the study period

	All patients (*n* = 485)	Alive (*n* = 326)	Dead (*n* = 159)	*p-*value
Demographics				
Age (years)	71·6 ± 16·3	68·4 ± 17·4	78·1 ± 11·5	**< 0·001**
Male sex [n(%)]	259 (53·4)	168 (51·5)	91 (57·3)	0·24
BMI (kg/m^2^)	26·3 (22·1–30·8)	26·7 (22·2–31·4)	25·5 (21·9–29·9)	0·13
BAME [n(%)]	73 (15·4)	59 (18·6)	14 (8·9)	**0·006**
Clinical Frailty Scale	5 (3–6)	3 (2–6)	6 (3–7)	**< 0·001**
Co-morbidities				
HFrEF [n(%)]	59 (12·2)	31 (9·5)	28 (17·6)	**0·010**
IHD [n(%)]	69 (14·2)	46 (14·1)	23 (14·5)	0·92
Hypertension [n(%)]	222 (45·8)	155 (47·5)	67 (42·1)	0·26
AF [n(%)]	97 (20·0)	52 (16·0)	45 (28·3)	**0·001**
Diabetes mellitus [n(%)]	147 (30·3)	90 (27·6)	57 (35·8)	0·064
Stroke/TIA [n(%)]	53 (10·9)	32 (9·8)	21 (13·2)	0·26
CKD [n(%)]	119 (24·5)	71 (21·8)	48 (30·2)	**0·043**
COPD [n(%)]	69 (14·2)	37 (11·3)	32 (20·1)	**0·009**
Malignancy [n(%)]	37 (7·6)	23 (7·1)	14 (8·8)	0·50
Medications				
ACEi [n(%)]	84 (17·3)	57 (17·5)	27 (17·0)	0·89
ARB [n(%)]	41 (8·5)	31 (9·5)	10 (6·3)	0·23
BB [n(%)]	113 (23·3)	75 (23·0)	38 (23·9)	0·83
CCB [n(%)]	80 (16·5)	57 (17·5)	23 (14·5)	0·40
Loop diuretic [n(%)]	74 (15·3)	47 (14·4)	27 (17·0)	0·46
MRA [n(%)]	18 (3·7)	12 (3·7)	6 (3·8)	0·96
Statin [n(%)]	200 (41·2)	136 (41·7)	64 (40·3)	0·76
Antiplatelet [n(%)]	115 (23·7)	75 (23·0)	40 (25·2)	0·60
Anticoagulant [n(%)]	67 (13·8)	38 (11·7)	29 (18·2)	**0·049**
Metformin [n(%)]	61 (12·6)	39 (12·0)	22 (13·8)	0·56
Sulphonylurea [n(%)]	23 (4·7)	18 (5·7)	5 (3·2)	0·23
Corticosteroid [n(%)]	24 (4·9)	14 (4·3)	10 (6·4)	0·34
Immunosuppression [n(%)]	22 (4·5)	13 (4·0)	9 (5·7)	0·41

*BMI* body mass index, *BAME* Black Asian and minority ethnic, *BMI* body mass index, *HFrEF* heart failure with reduced ejection fraction, *IHD* ischaemic heart disease, *AF* atrial fibrillation, *CKD* chronic kidney disease, *COPD* chronic obstructive pulmonary disease, *ACEi* angiotensin converting enzyme inhibitor, *ARB* angiotensin II receptor blocker, *BB* beta-adrenoceptor antagonist, *CCB* calcium channel blocker, *MRA* mineralocorticoid receptor antagonist

## Discussion

In this study, we present data regarding pre-emptive advanced care planning in patients admitted with SARS-CoV-2 infection during the COVID-19 pandemic according to national recommendations [17]. These decisions were made for the majority of hospitalised patients, who were often elderly, frail and multi-morbid. Advanced age, higher CFS and the accrued number of co-morbidities were independently associated with a decision to limit the ceiling of care below full intensive care-based treatment (level three). In contrast, only age and frailty were associated with death during hospitalisation, suggesting studies using ICU admission as a primary endpoint and/or measure of disease severity might be confounded by these decisions, which are infrequently available in care records.

### Addressing goals of care during the COVID-19 pandemic

Early reports from Wuhan [10, 18, 19] and Lombardy [2] highlighted the risks to patients due to demand for ICU care surpassing surge capacity [20, 21]. Considerable focus on preparedness has therefore included timely and patient-centred pre-emptive discussions, addressing advanced care planning in the setting of a potentially fatal illness which disproportionately effects the elderly and those with underlying health conditions. The priorities of such decisions were to avoid intensive and distressing treatments in patients who would not want to receive them and to manage patients appropriately according to the likelihood of benefit from intensive treatment [22].

Advanced care planning can be challenging for patients and healthcare professionals. It is essential that such discussions occur at an appropriate time and are framed within the individual patient’s beliefs and wishes [23]. At LTHT, establishing and documenting goals of care were assisted by the availability of the ReSPECT process [15]. This simple electronic documentation is standardised across care settings, and is recognised regionally by hospitals, primary care practices and ambulance services, and facilitates timely shared decision-making amongst patients, their next-of-kin and surrogate decision makers. Ceiling of care and CPR resuscitation decisions were documented in a high proportion of patients (84·3% and 93·0%) admitted with SARS-CoV-2 infection. Hence, LTHT is well-placed to report on the clinical application of this approach.

The COVID-19 pandemic continues to challenge healthcare systems globally, and has raised important ethical issues, particularly the need to prioritise access to limited resources to those with the greatest chance of survival and anticipated shorter recovery [20, 22]. The majority of our patients were considered inappropriate for escalation of care to an ICU setting, or for CPR. Although this could imply that decisions were influenced by a drive to protect resources, ICU and ward bed occupancy at LTHT was below surge capacity throughout the study period and a local database monitored and disseminated this information to treating teams daily. Hence, although a higher proportion of patients had the ReSPECT process completed during the peak months of the pandemic than would usually be expected, LTHT did not experience a severe shortage of ICU bed capacity, making it unlikely that the outcomes of these assessments were biased towards a particular decision by the capacity to provide care. The high rate of not-for-resuscitation and ceiling of care decisions therefore probably reflects the demographics and clinical characteristics of our patients who were often elderly, frail and multi-morbid. Furthermore, in a multivariable model adjusted for age and CFS, receipt of level two or three care were associated with an increased risk of death, reflecting baseline differences in disease severity in patients who were considered appropriate for intensive treatments. It is feasible, however, that a more challenging environment including the requirement for appropriate personal protective equipment prior to CPR, risks of transmission to healthcare professionals [24] and the limited effectiveness of cardiopulmonary resuscitation in the setting of COVID-19 [25] may have influenced these decisions.

### Demographic and clinical characteristics and their association with ceiling of care decisions

In response to the appreciation that a high proportion of patients admitted with SARS-CoV-2 infection were elderly and frail, and therefore potentially unlikely to benefit from intensive treatments, even in the absence of COVID-19 infection, on 20th March 2020 the National Institute for Health and Care Excellence (NICE) produced guidance that included the CFS as an adjunct to assist in discussions with patients and carers around advanced care planning and suitability for ICU care in the event of deterioration [17]. The CSF is a nine point scale which provides healthcare professionals with a simple screening tool for measuring frailty. The CSF has been validated in frail patients receiving ICU care, in which it reliably predicts outcomes [26, 27]. In our patients, frailty was a strong predictor of ceiling of care decisions, which is likely to reflect both the known poor prognosis in frail patients receiving ICU and also the aforementioned national recommendations. As would be expected, advanced age was strongly associated with these decisions, with patients aged over 85 years being far more likely to be deemed ineligible for level three care compared to those under 65.

BAME patients were more likely than White-European patients to be considered appropriate for level three care (OR 5·7, 95% CI 3·1–10·4, *p* < 0·001), however they were on average younger, had fewer co-morbidities and were less frail. In age-sex adjusted analysis, ethnicity was not associated with ceiling of care suggesting that these decisions are unlikely to be contributing to the worse outcomes in BAME patients. These observations are relevant to observational data from the UK and elsewhere with White-European patients being on average older.

### ICU admission per se is not a reliable marker of severe disease when advanced care planning is routine

The low rate of public testing for SARS-CoV-2 infection in many countries meant that studies have often been limited to hospitalised patients which have not considered the confounding effects of advanced care planning, such that a valid picture of risk factors for severe disease in elderly, frail and multi-morbid populations is unknown. Studies investigating risk factors in COVID-19 have often classified ICU admission as a marker of severe disease, either in recognition of poor outcomes in these patients or where mortality data were not yet available. For example, a population based study in the UK found that the receipt of an angiotensin-converting enzyme inhibitor (ACEi) or angiotensin-II-receptor blocker (ARB) were associated with a reduced risk (except in Black-African patients) of severe disease [9]. However, without accounting for the confounding effects of these decisions, admission to ICU or receipt of mechanical ventilation might be associated with *better* prognosis when compared with conservative management for patients in whom these treatments were considered inappropriate or futile [28]. Furthermore, these observations have not been confirmed in studies restricted to patients already on ICU in which death was the primary end-point [29, 30].

In our patients, prescription of ACEi (but not ARB) was associated with reduced likelihood of being considered appropriate for level three care, but was not associated with an increased risk of death during hospitalisation. These observations may be a result of biases introduced by pre-emptive ceiling of care decisions where those with co-morbidities are not considered for ICU care, and the relative risk of severe disease therefore appears less where this definition is applied. In our patients, admission to ICU was guided by these pre-emptive decisions in addition to severity of disease, however, the setting of care was not consistent for all patients. For example, some patients who were deemed inappropriate for mechanical ventilation received CPAP in an ICU setting, whilst others received these treatments in a ward or high-dependency unit.

A further paradox revealed by our data is that whilst patients deemed appropriate for level three care were more unwell at presentation, as evidenced by more abnormal laboratory values, chest radiography and physiological assessment, when compared to patients suitable for level one or two care, outcomes were favourable with the vast majority surviving until discharge. In our analysis, all those patients deemed appropriate for level three care who then died, did so following escalation of their care to an ICU setting and mechanical ventilation. Overall 14 (18·7%) of patients deemed eligible for level two and 61 (48·8%) of those deemed eligible for level three care were admitted to an ICU setting allowing for delivery of key treatments for COVID-19 of CPAP and mechanical ventilation. Including patients in whom ceiling of care decisions were not made, 38 (7·8%) patients received mechanical ventilation. This rate was low compared to earlier reports from Wuhan and Lombardy, but similar to contemporary reports from the UK [7]. Despite an average age of 57·8 ± 13·2 years, more than half of these patients had died at follow-up, with fewer than one in five having a successful discharge and, at the time of reporting, almost a quarter still in hospital. It might therefore be reasonable to regard mechanical ventilation as a marker of severe disease in patients who remain in hospital at the time of censorship, due to the poor prognosis in this group.

### Strengths and limitations

This is an analysis of a carefully characterised cohort of consecutively admitted patients with SARS-2-CoV infection in whom standardised documentation of ceiling of care decisions was routine and completed in the majority of cases. The principle limitations are inherent to the retrospective design and single centre setting, and our findings should be interpreted in light of this. Recommendations to assist treating teams making ceiling of care decisions were available [17], however these have not been validated in COVID-19, nor are there randomised data supporting their use. These decisions were not standardised, rather they were made between patients, their next-of-kin or surrogate decision makers following discussions regarding their goals of care. Whilst we recognise that there is a risk of undertreatment with this approach, it is also the case that undertreatment from a medical perspective may not equate to undertreatment from the perspective of patients or their relatives.

In our manuscript we show that individual co-morbidities were not associated with decisions to limit care, however accrued co-morbidities were. The inclusion of a validated co-morbidity index may have enriched our analysis of the impact of co-morbidities on ceiling of care decisions. An additional limitation is the low proportion of patients in this study who were BAME, although the proportion is in line with census data and representative of local demographics. BAME patients were younger and had fewer co-morbidities than White European patients making definitive conclusions regarding the impact of ethnicity on advanced care planning difficult. The availability of ICU beds [31], demographic and cultural differences between countries may limit the generalisability of our findings. The need for oxygen therapy is an imprecise outcome reflecting a wide range of disease severities, however is in line with other observational and interventional studies in COVID-19 [32]. Finally, the follow-up time is relatively short and outcomes data were therefore not available for patients who remained in hospital.

## Conclusions

To our knowledge, this study is the first to report ceiling of care and CPR decisions during the COVID-19 pandemic and their association with demographic and clinical characteristics. Decisions were made broadly in line with known predictors of poor outcomes in COVID-19, but with a focus on co-morbidities suggesting ICU admission might not a reliable endpoint for observational datasets aiming to explore risk factors of severe disease, with those at the greatest risk of death being the least likely to receive intensive treatments.

## Data Availability

The datasets generated and/or analysed during the current study are not publicly available due to the inclusion of potentially identifiable information but are available from the corresponding author upon reasonable request.

## Abbreviations

SARS-CoV-2: Acute respiratory syndrome coronavirus 2
WHO: World Health Organisation
COVID-19: Coronavirus disease 2019
ICU: Intensive care unit
RAAS: Renin-angiotensin-aldosterone system
CPR: Cardiopulmonary resuscitation
LTHT: Leeds Teaching Hospitals NHS Trust
ReSPECT: Recommended Summary Plan for Emergency Care and Treatment
BAME: Black-Asian-Minority-Ethnic
CKD: Chronic kidney disease
COPD: Chronic obstructive pulmonary disease
IHD: Ischaemic heart disease
HFrEF: Heart failure with reduced ejection fraction
TIA: Transient ischaemic attack
CFS: Clinical Frailty Scale
CPAP: Continuous positive airways pressure
OR: Odds ratio
CI: Confidence interval
